# The benefit of whole brain reirradiation in patients with multiple brain metastases

**DOI:** 10.1186/1748-717X-8-186

**Published:** 2013-07-24

**Authors:** Zerrin Ozgen, Beste M Atasoy, Aysegul Ucuncu Kefeli, Askin Seker, Faysal Dane, Ufuk Abacioglu

**Affiliations:** 1 MH-Marmara University Pendik Education and Research Hospital Radiation Oncology Clinic, Fevzi Cakmak mah, Mimar Sinan cad. No. 41 34899, Ust Kaynarca Pendik, Istanbul, Turkey; 2 Marmara University School of Medicine Department of Radiation Oncology, Istanbul, Turkey; 3 Marmara University School of Medicine Department of Neurosurgery, Istanbul, Turkey; 4 Marmara University School of Medicine Department of Internal Medicine Division of Medical Oncology, Istanbul, Turkey; 5 Neolife Medical Center Radiotherapy Clinic, Istanbul, Turkey

**Keywords:** Brain metastases, Cancer, Reirradiation, Whole brain radiotherapy

## Abstract

**Background:**

To assess the outcomes, symptom palliation and survival rates in patients who received repeat whole brain radiotherapy (WBRT).

**Methods:**

Twenty-eight patients who had progression of brain metastasis received a second course of WBRT. Univariate log-rank testing and multivariate Cox regression analysis were used to determine the factors for death among several variables (cumulative BED [BEDcumulative], primary tumor site, Karnofsky performance scale [KPS], previous SRS, number of metastases and absence of extracranial metastases). Correlations between variables and treatment response were evaluated with the Chi-squared test.

**Results:**

The median KPS was 60 (range 50 to 100) at the initiation of reirradiation. The median time interval between the two courses of WBRT was 9.5 months (range 3–27 months). The median doses of the first course and the second course of WBRT were 30 Gy (range 20 to 30 Gy) and 25 Gy (range 20 to 30 Gy), respectively. The mean BEDcumulative was 129.5 Gy (range 110 to 150 Gy). Severe or unexpected toxicity was not observed. Symptomatic response was detected in 39% of the patients. The median overall survival following reirradiation was 3 months (range 1 to 12 months, 95% CI 1.82-4.118). Survival was significantly better in responders (median 10 months, 95% CI 3.56-16.43) compared with non-responders (median 2 months, 95% CI 1.3-2.64) (p = 0.000). In multivariate analysis, patients that had lung cancer (p = 0.01), initial KPS ≥60 (p = 0.03) or longer intervals to reirradiation (p = 0.01) had significantly better survival rates.

**Conclusions:**

A careful second course of whole brain irradiation might provide a symptomatic and survival benefit in patients with good performance status and longer cranial progression-free intervals.

## Background

Although the exact incidence of brain metastasis is unknown, it is one of the most frequent manifestations in the daily practice of oncology [1,2]. The prognosis of these patients is usually poor. The median survival is 1 month in untreated patients and 4 to 6 months in treated patients [3,4]. Whole brain radiotherapy (WBRT) is the most common treatment modality and it prolongs survival in these patients. It has an effect on palliation and stabilizing cranial progression. In progressive situation, there are several options such as surgery and/or stereotactic radiosurgery (SRS) for the treatment of brain metastasis. These therapeutic interventions can be administered before and/or after WBRT. Moreover, adding SRS or surgical resection to WBRT may reveal a longer survival and a symptomatic control [5]. These treatments may also have an additional benefit in the selected patients such as younger age with good performance status, have less than four lesions and have no evidence of systemic disease [6]. Nevertheless, in cases of progression of the multiple metastases following any of these modalities whole brain reirradiation can be required, but, the risk of toxicity limits this large volume-based radiation therapy. Several factors i.e. the interval between the first and second of WBRT, total cumulative radiation dose and the fractionation scheme should be considered to avoid an excessive toxicity [7].

In this retrospective single center analysis, we aimed to assess whether there is both a benefit and an advantage for symptom palliations and survival in patients who received repeat WBRT.

## Methods

The current study has been approved by Ethics Committee in our faculty. A total of 28 patients were reirradiated due to progression of brain metastasis anywhere in the brain tissue, in the period between 2005 and 2013. Characteristics of the patients, diseases and treatments are provided in Table 1. One (4%) patient underwent resection for intracranial disease before the first course of WBRT. Five (18%) patients received SRS after the first course of WBRT, and 5 (18%) patients both underwent resection before and received SRS after the first course of WBRT. All SRS was performed in the Gamma Knife unit with a median of 14 Gy/single fraction (range 12 to 16 Gy). SRS was done median 11 months (range 4 to 14 months) following irradiation and second course of WBRT was performed median 8 months (range, 4 to 25 months) following SRS. Meanwhile, 17 (61%) patients did not receive any other treatment either before or after the first course of WBRT. Twenty-four (86%) patients had multiple metastases, and four (14%) patients had 3 to 4 metastases at the time of reirradiation. The biological effective dose (BED) was calculated using the linear quadratic model: (BED = nd (1 + d / [a/b])) in grays, where d = fraction dose (in grays), n = number of fractions, nd = D = total physical dose (in grays), and a/b is the ratio of 2 Gy [8]. The cumulative BED (BEDcumulative) was calculated by the addition of the BED of the first and second courses of WBRT.

**Table 1:** Characteristics of patients, diseases and treatments in the study group

**Characteristics**	
Age	Median 52 years old (range 36 to 68 years)
Sex	Male (11), Female (17)
Primary site of cancer	Breast (6), Lung (22)
Histology of primary disease	Invasive ductal cancer (breast); Non-small cell lung cancer (lung)
KPS before the first course of WBRT	Median 80 (range, 60–100)
KPS before the second course of WBRT	Median 60 (range, 50–100)
First course RT (Gy)	Median 30 Gy (range 20–30 Gy)
Second course RT (Gy)	Median 25 Gy (range 20–30 Gy)
BEDcumulative	Median 129.5 Gy (range 110–150 Gy)
Treatments	Surgery before first WBRT (1)
	SRS following first WBRT (5)
	Surgery before and SRS after first WBRT (5)
	None (17)

*WBRT* whole brain radiotherapy, *KPS* Karnofsky performance scale, *RT* radiotherapy, *BED* biological effective dose.

The median KPS was 60 (range 50 to 100) at the initiation of reirradiation. The most common neurological symptoms were as follows: 57% headache, 32% nausea, 25% weakness, 25% ataxia and 4% visual changes. At the time of the second course of WBRT, 16 (57%) patients had systemic disease. All of the patients were administered dexamethasone before and after reirradiation at a median dose of 8 mg (range, 4 to 16 mg).

Survival time was defined as the time from the diagnosis of brain metastasis (OS1) and from the start of the second course of WBRT (OS2) to the date of death. All of the survival curves were plotted using the Kaplan-Meier method. The median BEDcumulative dose, primary tumor site, median Karnofsky performance scale (KPS), previous SRS, number of metastases and absence of extracranial metastases were used as variables. A univariate log-rank test and multivariate Cox regression analysis were used to determine the factors for death among the variables. Treatment response related to variables was evaluated using the Chi-squared test.

## Results

The median dose of the first course of WBRT was 30 Gy at 3 Gy per fraction (range 20 to 30 Gy). Different fractionation schedules, such as 25 Gy (n = 21), 20 Gy (n = 5) and 30 Gy (n = 2) in 10 fractions, were used. The median time interval between the two courses of WBRT was 9.5 months (range 3 to 27 months). The median dose for the second course of WBRT was 25 Gy in 2 Gy per fraction (n = 21) (range 20 to 30 Gy). Other fractionation schedules were as follows: 20 Gy in 10 fractions (n = 4); 20 Gy in 5 fractions (n = 1); and 30 Gy in 10 fractions (n = 2). Therefore, the mean BEDcumulative was 129.5 Gy (range 110 to 150 Gy) (Table 1). Patients who received SRS had longer intervals than patients who did not (mean intervals, 14.9 ± 2.08 months vs. 10.15 ± 1.40 months, p = 0.47).

**Figure 1:**
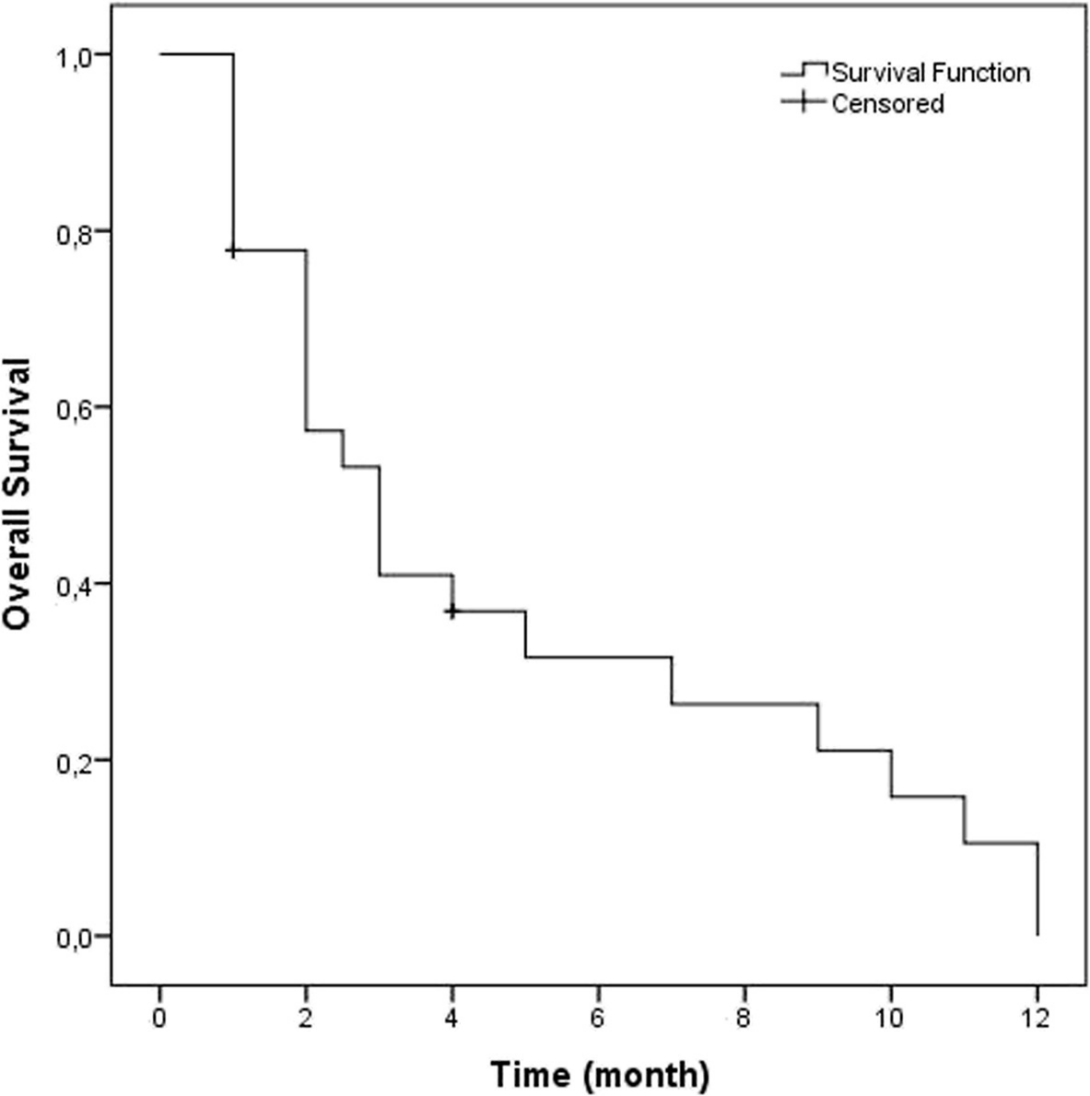
Overall survival following reirradiation in all patients.

**Figure 2:**
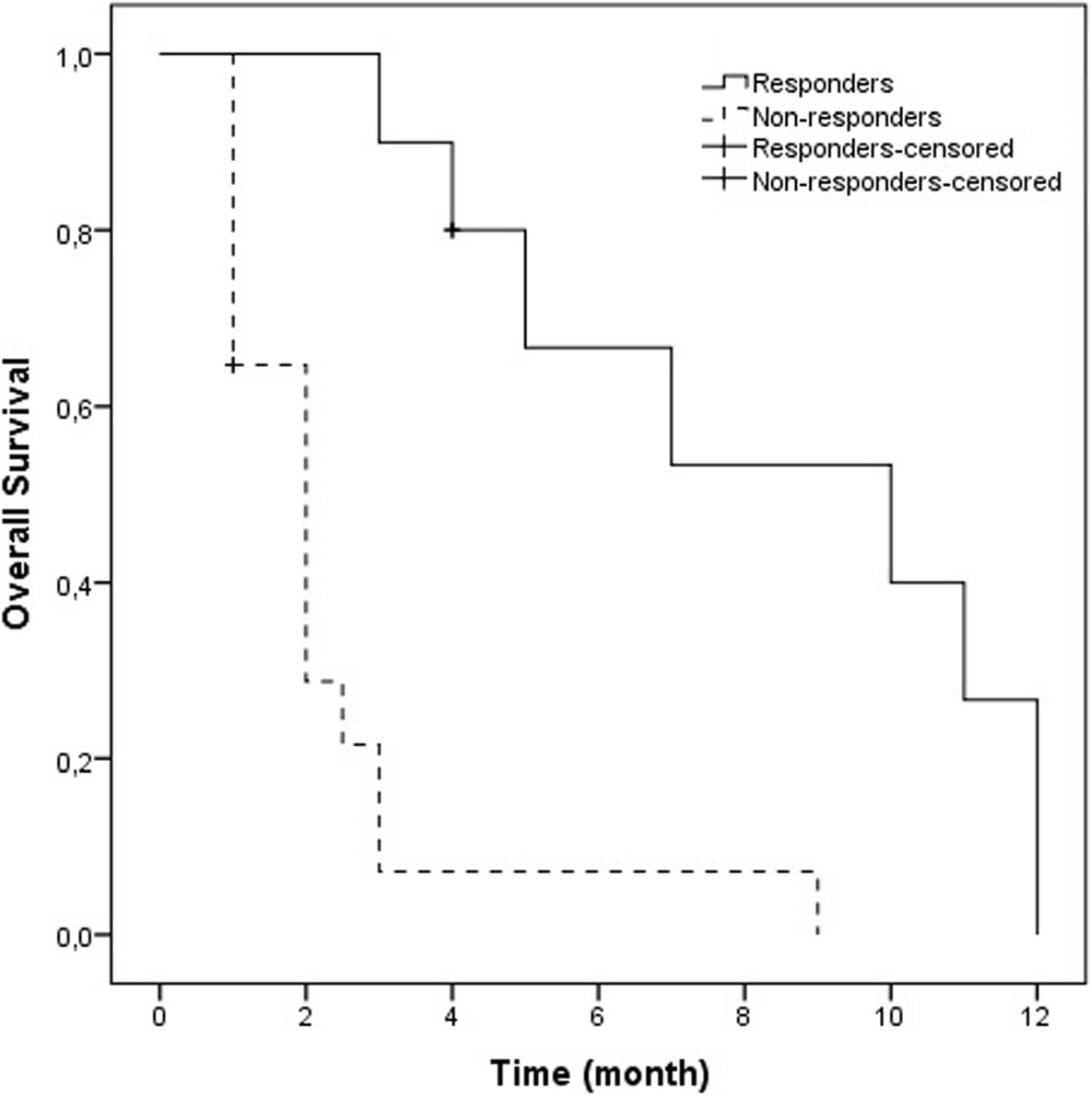
Overall survival according to responders (3 months) and non-responders (10 months) after reirradiation (p = 0.000).

Severe or unexpected toxicity was not observed. Symptomatic response was detected in 39% of patients, whereas the remainder of the patients had stable or progressive disease. The median OS1 was 12 months (range 5 to 28 months, 95% CI 10.13-13.86), and the OS2 was 3 months (range 1 to 12 months, 95% CI 1.82-4.118) (Figure 1). Following reirradiation, median survival was significantly better in responders (10 months, 95% CI 3.56-16.43) than in non-responders (2 months, 95% CI 1.3-2.64) (p = 0.000) (Figure 2). This result was independent of the presence of extracranial metastatic disease.

In univariate analysis, none of the factors showed any significance in terms of response to treatment (Table 2). In multivariate analysis, patients that had lung cancer (p = 0.01), had initial KPS ≥60 (p = 0.03) or had longer intervals to reirradiation (p = 0.01) had significantly longer survival (Table 3).

**Table 2:** Analysis of variables for response to the second course of WBRT

	**Responders (n)**	**Non responders (n)**	***p***
**Median dose (BEDcumulative)**			
≥129.5 Gy	6 (43%)	8 (57%)	
<129.5 Gy	5 (38%)	9 (62%)	0.50
**Primary diagnosis**			
Lung	10 (45%)	12 (55%)	
Breast	1 (16%)	5 (84%)	0.21
**Initial KPS**			
<60	4 (50%)	4 (50%)	
≥60	7 (54%)	13 (46%)	0.22
**SRS**			
(−)	8 (64%)	10 (36%)	
(+)	3 (30%)	7 (70%)	0.36
**Number of metastasis**			
<5	2 (66%)	1 (33%)	
≥5	9 (32%)	16 (68%)	0.33

*BED* biological effective dose, *KPS* Karnofsky performance scale, *SRS* stereotactic radiosurgery.

**Table 3:** Multivariate analysis for factors related to overall survival following reirradiation

**Factors (in favor of)**	***p***
Primary site (lung)	**0.01**
Pretreatment KPS (≥60)	**0.03**
Interval between two courses of WBRT (≥9.5 months)	**0.01**
SRS (application)	0.57
Extracranial metastasis (in control)	0.86
Number of metastasis in the brain (<5)	0.48
BEDcumulative (≥129.5 Gy)	0.62

*KPS* Karnofsky performance scale, *WBRT* whole brain radiotherapy, *SRS* stereotactic radiosurgery, *BED* biological effective dose.

## Discussion

There are several options, such as surgery and/or SRS, for patients that have a limited number of brain metastases in progression [5]. However, no consensus exists regarding the treatment for progressive multiple lesions in the brain. These patients can require a second course of whole brain irradiation, but the rationale for this treatment has not been well described. Not only treatment of symptomatic lesions but also the benefit of a survival advantage without any further toxicity are expected.

There have been more than a few articles in the literature describing whole brain reirradiation [9-14]. Some of these studies have reported comparable fractionation schemes and doses to those in our study [9-13] (Table 4). Despite different response rates between 27% and 80%, the median survival time in these studies was not longer than 5 months. In our study, the median survival was 3 months, and the response rate was 39%, which were very similar to the findings in the current literature. Thus, repeat radiotherapy could be a useful option in multiple intracranial progression. However, it is difficult to draw any conclusions about anti-edema therapy because almost all of the patients in our study received routine dexamethasone.

**Table 4:** Results of reirradiation studies in summary

	**Sadikov **[9]	**Hazuka **[10]	**Cooper **[11]	**Wong **[12]	**Son **[13]	**Our study**
	**n = 72**	**n = 44**	**n = 52**	**n = 86**	**n = 17**	**n = 28**
WBRT						
First course	20 Gy/5 fr	30 Gy/10 fr	30 Gy/10 fr	30 Gy/10 fr	35 Gy/14 fr	30 Gy/10 fr
Second course	25 Gy/10 fr	25 Gy/8 fr	25 Gy/10 fr	20 Gy/10 fr	21.6 Gy/12 fr	25 Gy/10 fr
Interval (median)	9.6 months	7.8 months	>4 months (mean)	7.6 months	15 months	9.5 months
Other treatments to the brain					SRS in 9 patients	SRS in 10 patients
					Surgery in 4 patients	
Response (%)	40	27	42	70	80	39
Survival after reirradiation (median)	4.1 months	2 months	4 months	4 months	5.2 months	3 months
						10 months (responders)
						3 months (nonresponders)

*WBRT* whole brain radiotherapy, *fr* fraction, *SRS* stereotactic radiosurgery.

The weak point of our study was that the side effects could not be reported in grades or in greater detail due to the retrospective nature of this study. Based on the follow-up notes and data, we can say that none of our patients suffered from severe neurotoxicity or died due to reirradiation. Because of the importance of toxicity, longer follow-up periods after the first course of WBRT should be expected by clinicians. SRS between two whole brain treatments might provide for longer intervals, as in our study. However, it did not demonstrate an advantage for survival in our study. In addition, we had four patients whose intervals were shorter than 6 months. These four patients died at 1, 2, 4 and 7 months after therapy due to disease progression. Although, whole brain parenchyma should only be irradiated in selected patients, because we believe that the dose per fraction may be kept at ≤2 Gy to avoid toxicity.

We could not find any positive correlation between dose increment and response in our patients in whom the cumulative BED was almost 130 Gy. Hence, we argued that increasing the dose might not help to improve the response or outcome, and 25 Gy in 10 fractions can therefore be recommended for whole brain reirradiation.

## Conclusions

Patients who have good performance status, have cranial progression-free intervals and who do not have progression of systemic disease might benefit from a second course of whole brain radiotherapy. Further reports on quality of life and toxicity analyses following reirradiation could help to determine the state of this treatment.

## Abbreviations

WBRT: Whole brain radiotherapy; BED: Biological effective dose; SRS: Stereotactic radiotherapy; KPS: Karnofsky performance scale.

## Competing interests

The authors have no personal conflicts of interest.

## Authors’ contributions

Conception and design were undertaken by ZO. Analysis and interpretation of data were performed by ZO, BMA, AUK and AS. Drafting of the article was performed by ZO and BMA. Critical revision of the article for important intellectual content was performed by ZO, BMA, FD and UA. Final approval of the version to be published was provided by ZO, BMA, FD and UA. All authors read and approved the final manuscript.
